# Differences in DNA Repair Capacity, Cell Death and Transcriptional Response after Irradiation between a Radiosensitive and a Radioresistant Cell Line

**DOI:** 10.1038/srep27043

**Published:** 2016-06-01

**Authors:** Mireia Borràs-Fresneda, Joan-Francesc Barquinero, Maria Gomolka, Sabine Hornhardt, Ute Rössler, Gemma Armengol, Leonardo Barrios

**Affiliations:** 1 Departament de Biologia Cel·lular, Fisiologia i Immunologia, Unitat de Biologia Cel·lular, Universitat Autònoma de Barcelona, Bellaterra, Catalonia, Spain; 2 Departament de Biologia Animal, Biologia Vegetal i Ecologia, Unitat d’Antropologia Biològica, Universitat Autònoma de Barcelona, Bellaterra, Catalonia, Spain; 3 Department Radiation Protection and Health, Federal Office for Radiation Protection, Neuherberg, Germany

## Abstract

Normal tissue toxicity after radiotherapy shows variability between patients, indicating inter-individual differences in radiosensitivity. Genetic variation probably contributes to these differences. The aim of the present study was to determine if two cell lines, one radiosensitive (RS) and another radioresistant (RR), showed differences in DNA repair capacity, cell viability, cell cycle progression and, in turn, if this response could be characterised by a differential gene expression profile at different post-irradiation times. After irradiation, the RS cell line showed a slower rate of γ-H2AX foci disappearance, a higher frequency of incomplete chromosomal aberrations, a reduced cell viability and a longer disturbance of the cell cycle when compared to the RR cell line. Moreover, a greater and prolonged transcriptional response after irradiation was induced in the RS cell line. Functional analysis showed that 24 h after irradiation genes involved in “DNA damage response”, “direct p53 effectors” and apoptosis were still differentially up-regulated in the RS cell line but not in the RR cell line. The two cell lines showed different response to IR and can be distinguished with cell-based assays and differential gene expression analysis. The results emphasise the importance to identify biomarkers of radiosensitivity for tailoring individualized radiotherapy protocols.

Radiotherapy is used to treat more than 50% of diagnosed cancers^1^,^2^. It is well known that, even when patients are treated with the same curative dose, normal tissue toxicity shows variability between patients, indicating inter-individual differences in the intrinsic radiosensitivity^1^,^3^,^4^,^5^. The mechanisms influencing intrinsic radiosensitivity still remain unclear and many factors may contribute to it, but it has been suggested that up to 80% of this variability could have a genetic basis^3^,^6^,^7^,^8^. In this sense, a better knowledge of these factors should lead to the development of *in vitro* predictive assays to identify radiosensitive individuals and, as a result, to establish individualised radiation therapy protocols^6^.

Although several promising biomarkers of cellular radiosensitivity have been tested, there is not enough evidence of their utility in clinical practice^9^. Among them, DNA damage markers, specifically those related with DNA double-strand breaks (DSBs), have been analysed and a certain level of association between cellular radiosensitivity tested *in vitro* and normal tissue reactions after radiotherapy has been observed^10^,^11^,^12^,^13^,^14^,^15^. On this basis, increased chromosomal aberration yields in peripheral blood lymphocytes and cell lines have been linked to radiosensitivity following *in vitro* ionising radiation (IR)^16^,^17^. It has also been described that the number of γ-H2AX foci is correlated with the number of radio-induced DSBs and that differences observed among individuals in the repair kinetics of γ-H2AX are possibly related with differences in radiosensitivity^18^,^19^,^20^.

Radiosensitivity is currently considered an inherited polygenic trait, dependent on the interaction of many genes^1^. In this regard, genetic variation probably contributes to inter-individual differences in developing undesirable side effects after radiotherapy. The analysis of gene expression profiles in individuals with different radiation toxicity will probably help to identify relevant candidate genes to predict these adverse side effects. Up to now, the great extent of transcriptomic studies have been based on microarray hybridisation technologies to measure gene expression changes from thousands of genes simultaneously, trying to identify biomarkers of radiation response^21^,^22^. Previous studies have described several gene expression signatures before and after irradiation in lymphocytes from patients or lymphoblastoid cell lines (LCLs) with different radiosensitivity^23^,^24^,^25^,^26^,^27^. The development of new high-throughput methods such as next-generation sequencing (NGS) technology, specifically using RNA sequencing analysis (RNA-seq)^28^,^29^, represents a promising tool to find biomarkers of radiosensitivity^30^,^31^,^32^.

Overall, most studies performed so far have tried to predict the radiation response using either cell-based assays or expression analysis but only few of them have used both approaches^24^,^33^. In a previous study, we observed differences in the levels of histone H2AX phosphorylation between a radiosensitive (RS) and a radioresistant (RR) cell line^34^. The aim of the present study was to determine if these differences could be related to DNA repair capacity, cell cycle progression or cell death and, in turn, if this response could be characterised by a differential gene expression profile at different post-irradiation times.

## Results

### After irradiation, a slower rate of γ-H2AX foci disappearance, a higher frequency of incomplete chromosome elements, a reduced cell viability and a higher cell cycle disturbance were observed in the RS cell line in comparison with the RR cell line

**Figure 1:**
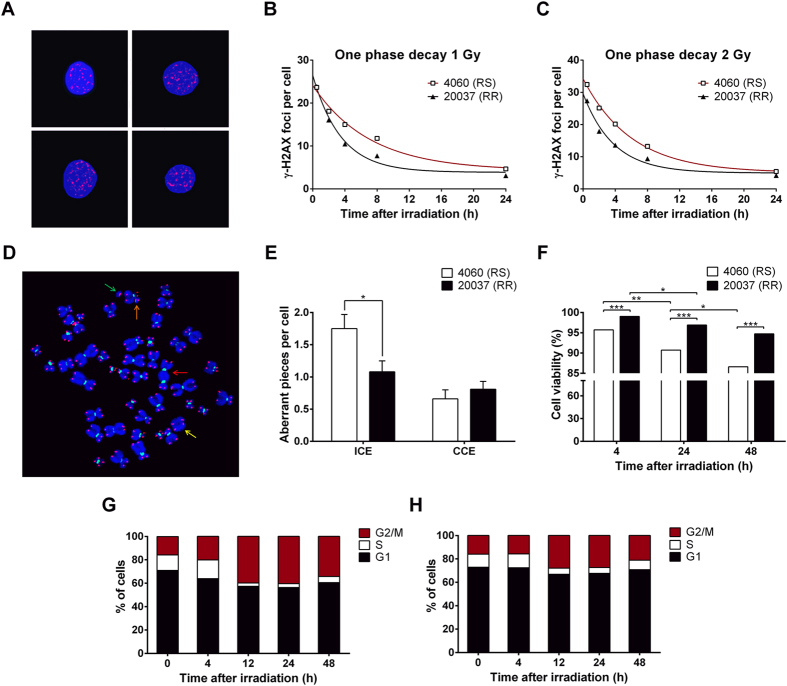
Cell-based assays in the RS (4060) and the RR (20037) cell line after irradiation. (**A**) MetaCyte images of γ-H2AX foci in the RS cell line 30 min after 2 Gy irradiation. (**B,C**) One phase decay curves of γ-H2AX foci disappearance after exposure to 1 Gy (**B**) and 2 Gy (**C**). γ-H2AX foci were scored in 800 cells for each particular experimental condition. (**D**) Metaphase image from the RS cell line analysed by pancentromeric and pantelomeric PNA-FISH. A dic(+/+) (red arrow), an ace(+/+) (yellow arrow), a chr(+/−) (orange arrow) and an ace(+/−) (green arrow) were observed. (**E**) Frequencies of ICE and CCE analysed by FISH. FISH analysis was carried out 24 h after 2 Gy irradiation. One hundred metaphases were analysed for each particular condition. Data were plotted as frequencies ± SE (error bars). Asterisk denotes significant differences (*p* < 0.05, Mann-Whitney test). (**F**) Cell viability of the RS and the RR cell line 4, 24 and 48 h after 2 Gy irradiation. Results were normalised to sham-irradiated cells and are expressed as mean percentage of viable cells from two independent experiments. A minimum of 1000 cells were analysed for each particular experimental condition. Asterisks denote significant differences (*p < 0.05; **p < 0.01; ***p < 0.001, z-test). (**G,H**) Cell cycle analysis by flow cytometry of the RS and the RR cell line before and 4, 12, 24 and 48 h after an exposure to 2 Gy irradiation. Results show the percentage of cells in G1, S and G2/M cell cycle phases. At least 20,000 cells were analysed for each particular experimental condition.

After 1 and 2 Gy irradiation, γ-H2AX foci induction and kinetics of their disappearance with post-irradiation time were assessed (Fig. 1A–C). At each analysis point, data sets from the two replicas were merged because no significant differences were observed. The maximum level of H2AX phosphorylation at both doses and for both cell lines was reached 30 min post-irradiation. The RS cell line showed significantly higher foci counts than the RR cell line for almost all the post-irradiation times tested (*p* < 0.01, Mann-Whitney test), except for 30 min after 1 Gy irradiation. To test if differences existed in the rate of γ-H2AX disappearance with post-irradiation time between both cell lines, data were fitted to a one phase decay non-linear regression curve (Fig. 1B,C). The results clearly showed a prolonged half-life of γ-H2AX foci in the RS cell line in comparison with the RR cell line, 4.9 vs. 2.4 h at 1 Gy and 4.1 vs. 2.6 h at 2 Gy.

Using pancentromeric and pantelomeric fluorescent *in situ* hybridization (FISH) probes, chromosome aberrations were scored after 2 Gy of irradiation (Fig. 1D, Supplementary Table S1). As can be seen in Fig. 1E, the frequency of incomplete chromosome elements (ICE), those without one or two telomeric signals, was higher in the RS cell line than in the RR cell line (*p* < 0.05, Mann-Whitney test). On the other hand, the frequency of complete chromosome elements (CCE) was higher for the RR cell line, but without statistical significance.

Results of cell viability after 2 Gy are presented in Fig. 1F. The results were plotted as the mean percentage of viable cells from two independent experiments. The percentage of viable cells after irradiation in the RS cell line was significantly lower than in the RR cell line, at 4, 24 and 48 h after irradiation (*p* < 0.001, z-test). A significant viability reduction between 4 and 24 h and between 24 and 48 h after irradiation was also observed in the RS cell line (*p* < 0.01 and *p* < 0.05, z-test) but only between 4 and 24 h after irradiation in the RR cell line (*p* < 0.05, z-test).

Cell cycle distribution was similar in both cell lines before irradiation (Fig. 1G,H). However, irradiation resulted in a higher disturbance of the cell cycle distribution in the RS cell line when compared with the RR cell line. For both cell lines, exposure to 2 Gy led to cell cycle arrest at both G1- and G2/M-phases, particularly at 12 and 24 h after irradiation, as showed by the decrease of S-phase fraction and the increase of G2/M. The G2/M fraction reached a maximum 24 h after irradiation, 40% and 27% in the RS and the RR cell line, respectively. A progressive return to basal distribution could be seen, much slower in the RS cell line than in the RR one.

### QuantSeq analysis revealed a greater and a prolonged differential expression of genes after irradiation in the RS cell line in comparison with the RR cell line

The expression of genes involved in radiation response was evaluated by QuantSeq methodology at different time points up to 14 days post-irradiation. The results obtained for both cell lines on irradiated versus sham-irradiated samples at 4 and 24 h and 14 days after irradiation are available in Supplementary Datasets S1, S3, S4, S5, S6. The sequencing data have been submitted to the public functional genomics data repository Gene Expression Omnibus (GEO)^35^,^36^ under the accession number GSE80207.

The analysis showed differences between the RS and the RR cell line in the number of differentially expressed (DE) genes following 2 Gy irradiation (Supplementary Table S2). At 4 h after irradiation, a greater transcriptional response was induced in the RS cell line (96 DE genes, 78 up-regulated and 18 down-regulated) when compared with the RR cell line (36 DE genes, 33 up-regulated and three down-regulated). At 24 h after irradiation, the same tendency was observed, the RS cell line still showed 58 DE genes (53 up-regulated and five down-regulated), whereas only seven genes were significantly up-regulated for the RR cell line. At 14 days after irradiation, there were no DE genes between the sham-irradiated samples and irradiated samples from both the RS and the RR cell line.

### Functional analysis showed that genes related with “DNA damage response”, “direct p53 effectors” and apoptosis were still differentially up-regulated 24 h after irradiation in the RS cell line but not in the RR cell line

Gene enrichment analysis using the ToppGene platform was performed. The set of differentially up-regulated genes for both cell lines 4 and 24 h after irradiation was enriched in pathways and biological processes known to be involved in radiation response, such as DNA damage response, cell cycle regulation and apoptosis (Tables 1, 2, 3, 4, [Table t3], [Table t3], [Table t4]).

In the RS cell line 4 h after irradiation the most enriched pathways were “direct p53 effectors” and “DNA damage response” (Table 1). Up to 24 h after irradiation, the same pathways were still enriched. In relation to biological processes at 4 h after irradiation, significant gene enrichment was found for “apoptotic process”, “cellular response to DNA damage stimulus” and “negative regulation of cell cycle” (Table 2). At 24 h after irradiation, the same biological processes were still enriched. For the down-regulated genes in the RS cell line, the only biological process enriched was “cell division” 4 h after irradiation (Table 2).

Regarding the differentially up-regulated genes of the RR cell line, the most enriched pathways were “direct p53 effectors” and “DNA damage response”, but only 4 h after irradiation (Table 3). In relation to biological processes, the most enriched one was “intrinsic apoptotic signaling pathway in response to DNA damage by p53 class mediator” at 4 h after irradiation (Table 4). For the down-regulated genes in the RR cell line, the only biological process enriched was “mitotic cell cycle process” but only 4 h after irradiation (Table 4).

**Figure 2:**
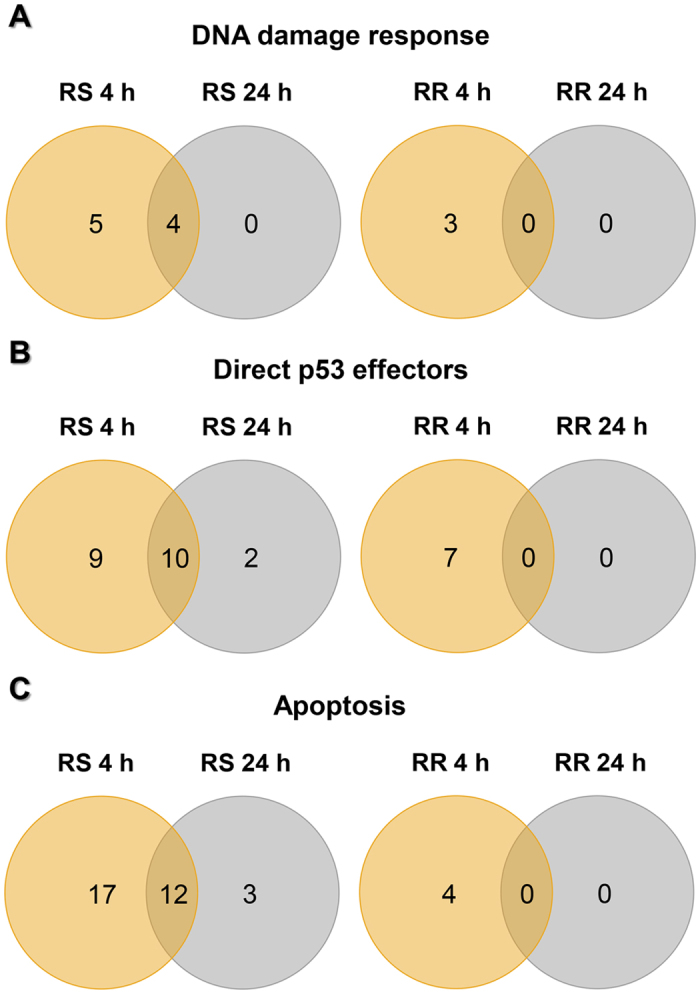
Venn diagrams of differentially up-regulated genes in the RS (4060) and the RR (20037) cell line, grouped according to the pathway or the biological process affected, 4 and 24 h after 2 Gy irradiation. (**A**) Differentially up-regulated genes related to the pathway “DNA damage response”. (**B**) Differentially up-regulated genes related to the pathway “direct 53 effectors”. (**C**) Differentially up-regulated genes related to biological processes of apoptosis.

To identify differences in up-regulated genes between both cell lines at different times after irradiation, Venn diagram analysis were performed for “DNA damage response”, “direct p53 effectors” and apoptosis (Fig. 2). As can be seen, most of the genes up-regulated in the RS cell line 24 h after irradiation were already present 4 h after irradiation. In contrast, the RR cell line did not show any DE genes 24 h after irradiation for any of the categories considered.

## Discussion

The tolerance of normal tissue is the limiting factor in the radiation dose applied for cancer treatments^5^,^37^. For this reason, efforts in this field have been focused on the development of assays that can predict the risk of developing acute normal tissue damage^9^,^37^,^38^. Understanding molecular mechanisms underlying radiation sensitivity will enable tailoring treatments to individual patients.

The differences in radiation sensitivity are often associated with a reduced ability to efficiently repair DSBs and/or activate the DNA damage response. For example, patients carrying mutations at *ATM* (Ataxia-Telangiectasia syndrome, A-T) or *BRCA1/2* are characterised by extreme radiosensitivity^39^,^40^,^41^. Variation in radiosensitivity can also be associated with differences in cell cycle regulation and apoptotic pathways^8^,^42^,^43^. In this sense, the two cell lines studied here are a good model given their previously reported different response to radiation^34^,^44^,^45^,^46^.

One of the initials steps in the DNA damage response is the phosphorylation of the histone H2AX. The RS cell line showed significantly higher foci counts and a slower rate of foci disappearance than the RR cell line. This is in agreement with studies on tumour cell lines, where a slower rate of γ-H2AX foci disappearance in RS cell lines has been described, when compared with RR tumour cell lines^19^,^47^. Moreover, a prolonged presence of γ-H2AX foci in cell lines with defective DSB repair after 3 Gy irradiation has been previously reported^43^.

The analysis of chromosome aberrations is a well established method to assess the damage produced by IR. In the present study, a significantly higher frequency of ICE in the RS cell line was observed in comparison with the RR cell line, indicating that more chromosome breaks failed to be rejoined in the RS cell line. It has been described that *ex vivo* irradiated lymphocytes from clinically radiosensitive patients showed higher frequencies of exchange-type aberrations and deletions than the non-radiosensitive patients^14^.

Cell death assays have the potential to evaluate individual radiosensitivity^1^,^48^,^49^. In our study, a significantly greater reduction in cell viability was observed in the RS cell line in comparison with the RR cell line at 4, 24 and 48 h post-irradiation. However, this reduction in viability was not very important, being around 10% and 14% in the RS cell line 24 and 48 h after irradiation, respectively. In the RR cell line, the reduction of viability was less important, around 3% and 5% at 24 and 48 h after irradiation, respectively. Although it is believed that a single unrepaired chromosome break could be sufficient for the induction of cell death^41^, it is possible that 2 Gy is an insufficient dose to trigger high levels of apoptosis. Some authors have described increased levels of apoptosis on LCLs irradiated at 5 Gy, but not at 2 Gy^50^,^51^,^52^. It has been reported that levels of apoptosis after 5 Gy increased from 24 to 48 h when determined by the sub-G1 method but decreased when determined by the TUNEL assay^52^. Moreover, it has been described that EBV immortalization can induce the expression of some anti-apoptotic EBV proteins^53^. These results indicate that apoptosis levels after irradiation are variable, depending on the type of cells analysed, the dose and the methodology used to measure apoptosis.

Cell cycle arrest in G2/M-phase was strongly induced by IR in the RS cell line compared to the RR cell line. This is in agreement with the results from other authors on LCLs after irradiation^24^,^50^,^51^. Cell cycle arrest is an important event in the response to radiation because it provides time for DSB repair, but there are inherent limitations. The G1/S checkpoint is slowly activated, allowing cells to enter S phase in the presence of unrepaired DSBs and other DNA damages^54^. The G2/M checkpoint is specially restrictive for cells with unrejoined chromosome aberrations^55^, but does not completely impede that cells with DSBs reach mitosis, because they can be released before DSB repair is complete^54^,^56^. Moreover, it has also been described that cell cycle progression takes longer for cells exhibiting slow repair kinetics^56^, although this cannot be always interpreted as a higher efficiency in DNA repair. In this regard, it has been reported that irradiated lymphocytes from *BCRA1* mutated heterozygotes showed a higher G2 delay but also a significantly higher frequency of G2 chromatid aberrations than normal controls, indicating that more damaged cells were able to pass the G2/M transition^57^. According to our results, in spite of the increased fraction of G2/M-phase cells at all post-irradiation times, the cell cycle arrest could not be fully effective in the RS cell line, since cells with more ICE could progress to M phase compared to the RR cell line.

Regarding gene expression, from the 12,349 genes analysed, less than 100 genes for each analysis were found to be DE. Moreover, a significant enrichment was observed for pathways or biological processes related to the response to IR. These differential gene expression profiles clearly demonstrate the capability of the QuantSeq methodology to discriminate between irradiated and unirradiated samples, but also to determine the changes in gene expression in relation to the time after exposure and the differences in the response between the RS and the RR cell line.

The different number of DE genes between the RS and the RR cell line, 4 and 24 h after irradiation, indicates that the RS cell line was transcriptionally more active than the RR cell line in response to IR. This is in agreement with previous results where more differentially regulated proteins were detected in the RS cell line than in the RR one after irradiation^45^. Furthermore, many of the DE genes reported here are related to DNA damage and repair, regulation of cell cycle and apoptosis, processes known to be involved in radiation response^22^,^24^,^25^,^27^,^58^,^59^,^60^,^61^.

Analysis of differential gene expression profiles at 24 h after irradiation revealed that no genes related to “DNA damage response”, “direct p53 effectors” and apoptosis were up-regulated in the RR cell line, whereas four, 12 and 15 genes were still DE in the RS cell line, respectively (Fig. 2). From these, four, ten and 12 genes, respectively, had been described as up-regulated after IR exposure by several authors^22^,^24^,^25^,^27^,^30^,^58^,^59^,^60^,^62^,^63^. Three of these genes (*RRM2B, CDKN1A* and *MDM2*) were common to the three categories. RRM2B is involved in cell cycle regulation and DNA repair, and its expression is p53-dependent^22^,^59^,^63^. *CDKN1A* expression is also p53-dependent and plays a key role in the negative regulation of cell cycle both at G1 and G2/M in response to DNA damage induced by IR^21^,^22^,^27^,^58^,^59^,^60^,^63^. MDM2 is a negative regulator of p53, and it is considered as a critical component of the cellular response to IR^22^,^24^,^25^,^27^,^63^,^64^.

Among other genes, *CDKN1A* and *MDM2* are also involved in “negative regulation of cell cycle”, a biological process significantly enriched only in the RS cell line 4 and 24 h after irradiation (Table 2). In the RR cell line *CDKN1A* was up-regulated at 4 h after irradiation but not at 24 h. Moreover, the RS cell line showed seven down-regulated genes related with the biological process “cell division” 4 h after irradiation, whereas the RR cell line only presented three down-regulated genes related to “mitotic cell cycle process”. All these repressed genes had been previously described as down-regulated in response to IR^22^,^24^,^25^,^27^,^50^,^58^,^59^,^60^,^62^,^63^. These results agree with the longer disturbance of the cell cycle distribution observed in the RS cell line versus the RR cell line. Moreover, the higher cell death observed in the RS cell line at 4, 24 and 48 h after irradiation is in agreement with the maintained up-regulation of apoptotic genes in this cell line.

The whole results obtained in the present work allow concluding that the two cell lines showed different response to IR, confirmed both by cell-based assays and the differential gene expression analysis. The RS cell line seemed to be less efficient in DNA repair (slow kinetics of foci disappearance and higher frequency of ICE), causing a longer disturbance of the cell cycle in comparison with the RR cell line. These characteristics were probably responsible for the reduced viability after irradiation observed in the RS cell line when compared with the RR cell line. Gene expression signatures corroborated these results, given the sustained expression of genes related with “DNA damage response”, “negative regulation of cell cycle” and apoptosis, which are still necessary up to 24 h after IR in the RS cell line, but not in the RR one.

## Methods

### Cell lines culture

The two cell lines used in the present study were generated by immortalisation of B-lymphocytes by Epstein-Barr-virus (EBV) infection of peripheral blood leukocytes obtained from lung cancer patients. The methods were carried out in “accordance” with approved guidelines and regulations at the Helmholtz Institution Munich and the Federal Office of Radiation Protection, Neuherberg. Informed consent was obtained from all subjects. The study and protocols were approved by the Ethical Committee of the *Bayerische Landesärztekammer*, Nr 99196, on January the 26th in the year 2000. Characterisation of the two cell lines by M-FISH was carried out before irradiation and no chromosome aberrations were observed. Their radiosensitivity was previously tested^34^,^44^,^45^,^46^. According to this, one cell line (4060-200) was defined as RS and the other (20037-200) as RR.

These LCLs were grown in suspension, at 37 °C in a 5% CO_2_ atmosphere, in Roswell Park Memorial Institute 1640 (RPMI-1640) medium (Biowest, Barcelona, Spain) supplemented with 15% fetal bovine serum (Life Technologies, Madrid, Spain), L-glutamine 2 mM (Biowest) and penicillin/streptomycin (100 U/mL and 100 μg/mL, respectively) (Biowest).

### Irradiation

The LCL cultures were irradiated in exponential growth phase at 1 and 2 Gy to study the kinetics of γ-H2AX foci and at 2 Gy for peptide nucleic acid (PNA) FISH analysis, cell death measurements, cell cycle analysis and RNA-seq. Sham-irradiated control cultures were also used in all analyses. Samples were irradiated with a ^137^Cs-irradiator (IBL437C, CIS Biointernational, Gif-sur-Yvette, France), at a dose rate from 4.87 to 4.84 Gy/min due to decay of source.

### Immunostaining and automated microscopic analysis of γ-H2AX foci

To assess the kinetics of γ-H2AX foci induction and disappearance following IR, cell cultures were maintained at 37 °C after irradiation for 10 and 30 min and for 2, 4, 8 and 24 h. After that, cell cultures were incubated on ice until processing. Immunostaining and automated microscopic analysis of γ-H2AX foci were performed as previously described^34^. Two replicas of 400 cells were analysed for each particular experimental condition.

Normality of foci distribution was tested with Kolmogorov-Smirnov with Lilliefors correction. Because compliance with normal distribution was not observed, nonparametric tests were used. Wilcoxon matched-pairs signed rank test was performed to assess if medians of signed ranks of the two replicas of γ-H2AX kinetics were different. Mann-Whitney test was used to compare foci counts.

### FISH with pancentromeric and pantelomeric PNA probes

After irradiation, cell lines were cultured at 37 °C for 24 h. Colcemid (Gibco, Life Technologies) was added 4 h before harvesting at a final concentration of 0.3 μg/mL. Harvesting was carried out by the standard hypotonic treatment (KCl 0.075 M at 37 °C for 5 min) followed by fixation with Carnoy’s solution (methanol:glacial acetic acid, 3:1, v/v).

FISH was performed according to a protocol previously described^55^. DAPI in Vectashield Mounting Medium (Vector Laboratories, Barcelona, Spain) was applied as counterstain. Slides were imaged with a Zeiss Axio Imager.Z2 microscope coupled to the Metafer 4 Slide Scanning System v3.10.2 (MetaSystems, Barcelona, Spain). Images were then analysed with the Isis software (MetaSystems).

To determine the percentage of first division mitoses, a parallel culture was carried out with the same conditions but adding bromodeoxyuridine (Sigma-Aldrich Química, Madrid, Spain) at a final concentration of 12 μg/mL following irradiation. The percentage of first division metaphases at 24 h post-irradiation was higher than 95% for both the RS and the RR cell line.

### FISH scoring criteria

For the FISH analyses only metaphases containing 46 centromeric signals and 92 telomeric signals were considered. Centromeric signals allowed detecting unequivocally dicentrics, rings and acentric fragments, and furthermore, telomeric signals allowed determining the completeness of each chromosome piece. Chromosomes lacking a telomeric signal at one or both ends were recorded as chr(+/−) or chr(−/−), respectively. The same criterion was applied for dicentric chromosomes [dic(+/−) and dic(−/−)] and acentric fragments [ace(+/−)]. Acentric fragments without telomeric signals were classified as interstitial deletions (ID) and centric ring chromosomes were classified as R. For comparisons, the aberrant chromosome pieces were grouped as CCE [dic(+/+), R and ace(+/+)], those with telomeric signals at both ends, or as ICE [chr(+/−), chr(−/−), dic(+/−), dic(−/−) and ace(+/−)], those without telomeric signals at one or both ends^55^. ID were not included in any category because of the difficulty to distinguish between an acentric fragment or an acentric ring when the ID is very small. One hundred metaphases were analysed for each particular experimental condition.

To compare the frequencies obtained in the FISH study between the RS and the RR cell line, Mann-Whitney test was applied.

### Cell death assay with Annexin V/Propidium iodide

To measure cell death after IR, the Annexin-V-FLUOS Staining Kit (Roche, Barcelona, Spain) was used following the manufacturer’s instructions. The cell lines were cultured at 37 °C for 4, 24 and 48 h after irradiation. Viable, apoptotic and necrotic (or late apoptotic) cells were scored by eye with an epifluorescence microscope BX51 (Olympus, Barcelona, Spain) using a 50× Plan objective.

Two independent experiments were performed analysing a minimum of 1000 cells for each particular experimental condition. To evaluate the radiation-induced increase of cell death, the obtained values after IR were normalized to those from sham-irradiated cells. A z-test was used to compare if the difference between proportions of viable and total cells in the RS and the RR cell line were significant.

### Cell cycle analysis

After 2 Gy irradiation cells were incubated at 37 °C for 4, 12, 24 and 48 h. Sham-irradiated controls were also used. Cells were centrifuged at 250 g for 5 min, washed in phosphate buffered saline and fixed in 70% ethanol at least for 2 h at −20 °C. Then, cells were incubated for 15 min at 37 °C with a staining solution containing propidium iodide (10 μg/mL) (Molecular Probes, Eugene, OR, USA), 0.1% TritonX100 (Sigma-Aldrich) and DNase-free RNase A (100 μg/mL) (Sigma-Aldrich) dissolved in 1× PBS. Cell cycle analysis was performed using a BD FACSCanto flow cytometer and BD FACSDiva software v7.0 (BD Biosciences, San Jose, CA, USA). At least 10,000 cells per replica were analysed and two replicas were performed at each time point.

### RNA extraction

To study the differential gene expression, irradiated (2 Gy) and sham-irradiated cell cultures of the RS and the RR cell line were incubated at 37 °C for 4 and 24 h and 14 days. After that, total RNA extraction was carried out with the SPLIT RNA Extraction kit (Lexogen Gmbh, Vienna, Austria) according to the manufacturer’s instructions. RNA concentration and purity for each sample were verified with a Nanodrop ND-100 spectrophotometer (Thermo Fisher Scientific, Waltham, MA, USA). The ratios of absorbance 260/280 nm and 260/230 nm were ∼2 for all the samples, and RNA concentration was ∼200 ng/μL per sample. RNA integrity was determined with Agilent 2100 Bioanalyzer (Agilent Technologies, Santa Clara, CA, USA). RNA Integrity Number (RIN) values close to 10 were obtained, indicative of high quality RNA samples. Pure RNA samples were stored at −80 °C until cDNA libraries were prepared.

### Library generation and sequencing

QuantSeq technology has been used as alternative to microarrays and conventional RNA-seq, in order to measure gene expression. cDNA libraries for each sample were generated at Functional Genomics Core (IRB Barcelona, Barcelona, Spain) from 500 ng of total RNA using the Quantseq 3′ mRNA-Seq Library Prep kit for Illumina (Lexogen) following the manufacturer’s instructions^65^. QuantSeq generates highly strand-specific NGS libraries close to the 3′ end of polyadenylated RNA. Briefly, the first cDNA strand is generated through reverse transcription initiated by oligodT priming. The synthesis of the second cDNA strand is performed by random priming, in a manner that DNA polymerase is efficiently stopped when reaching the next hybridised random primer, so only the fragment closest to the 3′ end gets extended until the end and gets both adapter sequences necessary for PCR amplification.

During library amplification (12 cycles of PCR), standard external barcodes were ligated to allow for multiplex sequencing. After PCR amplification, the libraries were size-selected with Agencourt AMPure XP magnetic beads (Beckman Coulter, Brea, CA, USA). Libraries were quantified by QuBIT (Life Technologies) and their average size of ∼250 bp was determined using a High Sensitivity DNA chip on an Agilent 2100 Bioanalyzer (Agilent Technologies).

Libraries were then sequenced (50 bp single read) by the NGS unit of Campus Science Support Facilities GmbH (CSF, Vienna, Austria) using an Illumina Hiseq2500 sequencer. Quality control analyses on the resulting bam sequencing files were performed using FastQC tool (Babraham Bioinformatics, Cambridgeshire, UK).

### Sequence alignment

Due to non-specific hybridisation of the random primer used to synthesise the second strand of cDNA, a trimming of the first nucleotides of the reads was performed before sequence alignment in order to improve the percentage of mapped reads (parameters available in www.lexogen.com/quantseq-data-analysis). Most sequences were originated from the last exon and the 3′ untranslated region (UTR). Sequence reads were aligned onto the human genome reference sequence (hg19) using the TopHat2 splice-junction mapper, version 2.1.0^66^.

### Quantification of reads and differential expression analysis with edgeR

As QuantSeq generates only one fragment per transcript, length normalization was not required and gene expression quantification was read-count based. Mapped reads were categorised with htseq-count (HTSeq version 0.6.1) directly linking the number of reads mapped to a gene to its expression^65^,^67^. Default settings were applied with the exception of the overlap resolution mode, where the intersection-nonempty mode was used, since this mode counts also the reads with incomplete annotation.

The package edgeR version 3.10.2 was used to identify DE genes^68^. Following the procedures described in^69^ and in the edgeR documentation (https://bioconductor.org), count files from htseq-count were loaded into R and normalised using the default method for edgeR (trimmed mean of M values, or TMM). To consider the multiple factors of our experimental design (two different cell lines, control and irradiated samples, different times of analysis and different replicas), a matrix was designed. Dispersion values were estimated using the Cox-Reid profile-adjusted likelihood method. Then, the generalised linear model likelihood ratio test was used to determine differential expression. Fold-changes were reported as the log (base 2) of normalised count abundance of the irradiated samples divided by count abundance for the sham-irradiated samples. P values reported for each gene were then corrected for multiple comparisons using Benjamini & Hochberg (B&H) false discovery rate (FDR)^70^.

### Functional analysis

To identify pathways and biological processes affected in response to IR, gene list enrichment analysis was performed using ToppGene suite (https://toppgene.cchmc.org)^71^. The resulting lists (applying a 0.05 FDR cut-off) of up-regulated and down-regulated genes from edgeR were then exported to the ToppFun application of the ToppGene platform.

Other platforms such as DAVID and PANTHER (data not shown), as well as GOseq package (version 1.20.0)^72^ (results are available in Supplementary Dataset S7) were used to perform functional analysis and very similar results were obtained.

## Additional Information

**How to cite this article**: Borràs-Fresneda, M. *et al*. Differences in DNA Repair Capacity, Cell Death and Transcriptional Response after Irradiation between a Radiosensitive and a Radioresistant Cell Line. *Sci. Rep*. **6**, 27043; doi: 10.1038/srep27043 (2016).

## Supplementary Material

Supplementary Information

Supplementary Dataset S1

Supplementary Dataset S2

Supplementary Dataset S3

Supplementary Dataset S4

Supplementary Dataset S5

Supplementary Dataset S6

Supplementary Dataset S7

## Figures and Tables

**Table 1:** Pathway analysis of DE genes in the RS (4060) cell line 4 and 24 h after 2 Gy irradiation.

Pathway	P value^a^	Number of genes	Gene list
Up-regulated			
*4 h*			
direct p53 effectors	7.79E-21	19	*BBC3, C12orf5, CDKN1A, DDB2, EPHA2, FAS, FDXR, GADD45A, GDF15, LIF, MDM2, PLK3, PRKAB1, RPS27L, RRM2B, SESN1, TNFRSF10B, TNFRSF10D, TRIAP1*
DNA damage response	4.66E-09	9	*BBC3, CDKN1A, DDB2, FAS, GADD45A, MDM2, RRM2B, SESN1, TNFRSF10B*
*24 h*			
direct p53 effectors	1.21E-12	12	*C12orf5, CDKN1A, FDXR, LIF, MDM2, RPS27L, RRM2B, SESN1, TNFRSF10D, TP53I3, TRIAP1, ZNF385A*
DNA damage response	6.82E-03	4	*CDKN1A, MDM2, RRM2B, SESN1*
Down-regulated			
*4 h*			
PLK1 signaling events	1.31E-08	5	*AURKA, CCNB1, CDC20, KIF20A, PLK1*
regulation of mitotic cell cycle	1.37E-05	4	*AURKA, CCNB1, CDC20, PLK1*

^a^*p* values were corrected for multiple comparisons using the FDR (B&H).

**Table 2:** Enriched biological processes of DE genes in the RS (4060) cell line 4 and 24 h after 2 Gy irradiation.

GO category	Category name	P value^a^	Number of genes	Gene list
Up-regulated				
*4 h*				
GO:0006915	apoptotic process	5.99E-07	29	*AEN, BBC3, BLOC1S2, C12orf5, CD70, CDKN1A, DRAM1, EDA2R, EPHA2, FAS, FHL2, FOSL1, GADD45A, GDF15, GLS2, MDM2, NOTCH1, NTN1, PHLDA3, PLK2, PLK3, PXT1, RPS27L, RRM2B, TNFRSF10B, TNFRSF10D, TNFSF4, TNFSF9, TRIAP1*
GO:0006974	cellular response to DNA damage stimulus	1.09E-05	17	*AEN, BBC3, C12orf5, CDKN1A, DDB2, E2F7, EPHA2, GADD45A, MDM2, PHLDA3, PLK3, POLH, RPS27L, RRM2B, SESN1, TRIAP1, XPC*
GO:0045786	negative regulation of cell cycle	2.47E-04	12	*CDKN1A, E2F7, GADD45A, LIF, MDM2, PLK2, PLK3, PRKAB1, RPS27L, SESN1, TRIAP1, XPC*
*24 h*				
GO:0006915	apoptotic process	1.16E-02	15	*ARHGEF3, C12orf5, CDKN1A, EDA2R, GLS2, MAP2K6, MDM2, PHLDA3, PXT1, RPS27L, RRM2B, TNFRSF10D, TNFSF4, TRIAP1, ZNF385A*
GO:0006974	cellular response to DNA damage stimulus	5.32E-04	12	*C12orf5, CDKN1A, E2F7, MAP2K6, MDM2, PHLDA3, RPS27L, RRM2B, SESN1, SPATA18, TRIAP1, ZNF385A*
GO:0045786	negative regulation of cell cycle	6.02E-03	8	*CDKN1A, E2F7, LIF, MAP2K6, MDM2, RPS27L, SESN1, TRIAP1*
Down-regulated				
*4 h*				
GO:0051301	cell division	1.02E-04	7	*AURKA, CCNB1, CCNF, CDC20, KIF20A, PLK1, PSRC1*

^a^*p* values were corrected for multiple comparisons using the FDR (B&H).

**Table 3 t3:** Pathway analysis of DE genes in the RR (20037) cell line 4 h after 2 Gy irradiation.

Pathway	P value^a^	Number of genes	Gene list
Up-regulated			
*4 h*			
direct p53 effectors	1.73E-06	7	*BBC3, CDKN1A, FDXR, GDF15, LIF, SESN1, ZNF385A*
DNA damage response	2.88E-02	3	*BBC3, CDKN1A, SESN1*

^a^*p* values were corrected for multiple comparisons using the FDR (B&H).

**Table 4 t4:** Enriched biological processes of DE genes in the RR (20037) cell line 4 h after 2 Gy irradiation.

GO category	Category name	P value^a^	Number of genes	Gene list
Up-regulated				
*4 h*				
GO:0042771	intrinsic apoptotic signaling pathway in response to DNA damage by p53 class mediator	1.47E-03	4	*BBC3, CDKN1A, PHLDA3, ZNF385A*
Down-regulated				
*4 h*				
GO:1903047	mitotic cell cycle process	4.32E-03	3	*CCNB1, GPSM2, NEK2*

^a^*p* values were corrected for multiple comparisons using the FDR (B&H).
