# Supplementary material for: Differences in DNA Repair Capacity, Cell Death and Transcriptional Response after Irradiation between a Radiosensitive and a Radioresistant Cell Line
Source: Sci Rep. 2016 Jun 1;6:27043. doi: 10.1038/srep27043 (PMC4887990; doi:10.1038/srep27043)
Supplement: Supplementary Information [file srep27043-s1.pdf]

SUPPLEMENTARY INFORMATION AND DATASET LEGENDS

TITLE: Differences in DNA Repair Capacity, Cell Death and Transcriptional Response after Irradiation between a Radiosensitive and a Radioresistant Cell Line

AUTHORS LIST: Mireia Borràs-Fresneda, Joan-Francesc Barquinero, Maria Gomolka, Sabine Hornhardt, Ute Rössler, Gemma Armengol and Leonardo Barrios

SUPPLEMENTARY INFORMATION

Supplementary Table S1. Chromosome analysis by FISH with pancentromeric and pantelomeric probes 24 h after 2 Gy irradiation

	Cell line	
	**4060 (RS)**	**20037 (RR)**
Incomplete chromosome elements (ICE)		
chr (+/-)	69	45
chr (-/-)	11	8
ace (+/-)	95	55
dic (+/-)	0	0
dic (-/-)	0	0
**Total ICE (frequency ± SE)**	175 (1.75 ± 0.22)*	108 (1.08 ± 0.17)
Complete chromosome elements (CCE)		
dic (+/+)	30	39
tri (+/+)	2	0
R	1	0
ace (+/+)	33	42
**Total CCE (frequency ± SE)**	66 (0.66 ± 0.14)	81 (0.81 ± 0.12)
***Interstitial deletions* (frequency ± SE)**	14 (0.14 ± 0.04)	7 (0.07 ± 0.03)

* p<0.05, significant differences obtained with Mann-Whitney test.

Supplementary Table S2. Differentially up- and down-regulated genes after 4 and 24 h and 14 days after 2 Gy irradiation

	Post-irradiation time	DE genes[a]	Up-regulated genes	Down-regulated genes
4060 (RS)	4 h	96	78	18
	24 h	58	53	5
	14 days	0	0	0
20037 (RR)	4 h	36	33	3
	24 h	7	7	0
	14 days	0	0	0

[a]After applying a 0.05 FDR cut-off.

SUPPLEMENTARY DATASETS

Supplementary Dataset S1. Complete differential expression results for the RS cell line 4 h after 2 Gy irradiation. Genes are ordered by their FDR values.

Supplementary Dataset S2. Complete differential expression results for the RR cell line 4 h after 2 Gy irradiation. Genes are ordered by their FDR values.

Supplementary Dataset S3. Complete differential expression results for the RS cell line 24 h after 2 Gy irradiation. Genes are ordered by their FDR values.

Supplementary Dataset S4. Complete differential expression results for the RR cell line 24 h after 2 Gy irradiation. Genes are ordered by their FDR values.

Supplementary Dataset S5. Complete differential expression results for the RS cell line 14 days after 2 Gy irradiation. Genes are ordered by their FDR values.

Supplementary Dataset S6. Complete differential expression results for the RR cell line 14 days after 2 Gy irradiation. Genes are ordered by their FDR values.

Supplementary Dataset S7. Functional analysis of differential expression results with GOseq for the RS cell line 4 and 24 h after 2 Gy irradiation. GO categories are ordered by the p value of the overrepresented ones.
